# Religiosity, Burnout, Depression, and Anxiety in a University Setting During COVID-19 in the USA: A Cross-Sectional Study

**DOI:** 10.1007/s10943-026-02689-0

**Published:** 2026-05-28

**Authors:** Abebaw Mengistu Yohannes, Harold G. Koenig, Candice R. Williams, Andrea N. Bowens

**Affiliations:** 1 Department of Physical Therapy, 372 School of Health Professions, University of Alabama at Birmingham, Birmingham, AL 35294 USA; 2 Allergy and Critical Care Medicine and Lung Health Center, University of Alabama at Birmingham, Birmingham, AL USA; 3 Departments of Psychiatry and Medicine, Duke University, Durham, NC USA; 4 Department of Medicine, King Abdulaziz University, Jeddah, Saudi Arabia; 5 Department of Criminal Justice, Sociology and Ethnic Studies, School of Education and Behavioral Sciences, Azusa Pacific University, Azusa, CA USA

**Keywords:** Depression, Anxiety, Religiosity, Burnout and COVID-19

## Abstract

The COVID-19 pandemic led to increased psychological burdens in all age groups. Limited literature has examined the relationship between religiosity, burnout, and mental health. We examined the relationship of religiosity to burnout, depression, and anxiety in a university setting. A questionnaire was emailed to all faculty, staff, and students at a single, faith-based university in the USA between November 2021 and April 2022. The survey contained 3-validated scales: the 16-item Oldenburg Burnout Inventory (OLBI), the 14-item Hospital Anxiety and Depression (HADS), and the 5-item Duke University Religion Index [which assesses organizational religious activity (ORA), non-organized religious activity (NORA), and intrinsic religiosity (IR)]. One hundred twenty-five questionnaires were completed by 17 faculty, 18 staff, and 90 students. The respondents’ mean age was 30.0 (SD = 12.3) years, with 69% female. Prevalence of depression was 41% (HADS-depression ≥ 8), anxiety was 62% (HADS-anxiety ≥ 8), and total burnout was 53% (OLBI mean > 2.18). Sixty-one (49%) participants attended religious services once or more per week, and 46 (37%) spent time in private religious activities daily. The odds ratio (OR) with (95% confidence interval, CI) for ORA with depression was 0.67 (0.53–0.86) and with anxiety was 0.78 (0.61–0.99). The OR for NORA with depression was 0.78 (0.61–0.99) and with anxiety was 0.77 (0.61–0.99). The OR for IR with depression was 0.79 (0.70–0.91) and with anxiety was 0.83 (0.73–0.96). Burnout was associated with both depression (OR = 10.08, 95% CI = 2.23–45.70) and anxiety (OR = 11.53, 95% CI = 2.31–57.47). Our findings showed that over 40% of participants experienced clinically significant depression, 60% experienced anxiety, and over 50% experienced burnout. Religiosity was associated with lower levels of depression and anxiety.

## Introduction

The COVID-19 pandemic disrupted university operations and shifted instruction to remote formats worldwide (UNESCO, 2021; U.S. Department of Education, 2022). The general population experienced pandemic-related psychological burdens, such as increased levels of anxiety, depression, and stress (Kupcova et al., 2023). University students were particularly vulnerable, reporting even greater psychological distress attributable to significant alterations in their academic and social lives (Browning et al., 2021; Chen & Lucock, 2022; Lee et al., 2021; Machado et al., 2023; Wong Aitken et al., 2024). In a survey of US college students, more than 80% of respondents reported an increase in symptoms of at least one of three mental health conditions such as anxiety, depression, or loneliness (Lee et al., 2021). Students in Europe also reported negative impacts on their mental and physical health during the first months of the pandemic (Chen & Lucock, 2022; Machado et al., 2023). Specifically, 36–49% of survey respondents in Portugal scored in the mild to extreme ranges of depression, anxiety, or stress, and 33% perceived worse health compared to pre-pandemic (Machado et al., 2023). One university in the UK, reported that more than 50% of students scored in the clinically significant ranges for anxiety and depression (Chen & Lucock, 2022).

Several studies have examined characteristics related to university students’ mental and physical health during the pandemic. A systematic review by Wong Aitken et al. (2024) identified academic stressors related to students’ psychological well-being. These stressors included challenges with adapting to education in isolation and issues related to virtual education, including deficits in learning resources (i.e., equipment, internet connectivity, and study space) and poor academic-life balance due to social isolation and decreased leisure activities. In addition to adapting their learning methods, students changed their lifestyle (Browning et al., 2021; Chen & Lucock, 2022; Lee et al., 2021; Machado et al., 2023). Lifestyle factors associated with higher levels of psychological impact included worsened interpersonal relationships, less exercise, more tobacco use, and spending eight or more hours on screens (Browning et al., 2021; Chen & Lucock, 2022). Lastly, researchers identified several sociodemographic factors significantly associated with higher levels of distress, worry, anxiety, or depression (Browning et al., 2021; Chen & Lucock, 2022). These factors included identifying as female, non-Hispanic Asian, younger between the ages of 18–24 years, and having a below-average family income.

Similar to students, the shift to remote operations globally impacted college and university faculty. Faculty have likewise reported higher levels of burnout and reduced mental and physical well-being during the pandemic (Almhdawi et al., 2021; Constantin et al., 2024; Rahman et al., 2024; Smith et al., 2022; Velez-Cruz & Holstun, 2022). Higher-education faculty in the USA feeling very to extremely stressed doubled from 32% in 2019 to 69% in 2020 during the pandemic (Chronicle of Higher Education, 2020). Psychological distress, anxiety, depression, and burnout experienced by faculty correlated with workloads, job insecurity, lack of institutional support, difficulty adapting to remote teaching, and providing emotional support to students (Almhdawi et al., 2021; Constantin et al., 2024; Rahman et al., 2024; Santiago et al., 2023; Chronicle of Higher Education, 2020). Like students, there were disparate impacts on faculty identifying with certain groups, most notably women, mid-career researchers, younger ages, and those living alone (Gewin, 2021; Rahman et al., 2024; Santiago et al., 2023; Smith et al., 2022; Chronicle of Higher Education, 2020).

In response to pandemic-related challenges, university faculty and students adopted various self-care and coping activities to manage their health and well-being. Faculty engaging in self-care during the pandemic, especially physical and emotional self-care, experienced higher compassion satisfaction and lower levels of burnout (Velez-Cruz & Holstun, 2022). The most common self-care activities reported by students were exercise or physical activity, mindfulness practices, and the use of digital health apps (Lee et al., 2021; Machado et al., 2023).^.^ Additionally, students employed positive coping strategies, including planning and acceptance, but also maladaptive strategies including self-blame and venting (Machado et al., 2023). Among the many coping mechanisms utilized, religion was the least frequently utilized adaptive response.

Researchers have previously evaluated both intrinsic and extrinsic forms of religiosity for their effects on health and well-being. Intrinsic religiosity, characterized by the integration of religion into one’s life and personal relationship with the sacred, demonstrated positive impacts on mental health and reduced symptoms of depression and anxiety (Campos et al., 2020; Hoogeveen et al., 2022; Koenig et al., 2020; Koenig & Büssing, 2010; Stewart et al., 2019; Taunay et al., 2012). During the COVID-19 pandemic, higher levels of intrinsic religiosity and regular religious service attendance were associated with lower levels of stress, anxiety, and depression in US adults and, specifically, university students (DeRossett et al., 2021; Jansen et al., 2010; Joseph et al., 2023). In contrast, extrinsic religiosity—which is defined as, using religiosity as a means to another non-religious end, such as social status, sociability, comfort, or security (Hoogeveen et al., 2022; Koenig & Büssing, 2010; Taunay et al., 2012)—and negative religious coping may lack the mitigating effects or even contribute to poorer psychological outcomes (Koenig et al., 2020; Rosmarin & Koenig, 2020; Weber & Pargament, 2014). During the pandemic, researchers noted a significant association between negative religious coping, defined as internal conflict with the divine or difficulty in finding meaning (DeRossett et al., 2021; Hoogeveen et al., 2022) and COVID-related anxiety (DeRossett et al., 2021). These findings highlight the complex relationship between religiosity and mental health, suggesting that the impact of religious beliefs and practices on individuals’ well-being may depend on the type of religious coping (DeRossett et al., 2021; Jansen et al., 2010).

Prior studies have noted elevated levels of anxiety, depression, and burnout among university students and faculty during the COVID-19 pandemic (Chen & Lucock, 2022; Kupcova et al., 2023; Lee et al., 2021; Lipson et al., 2022; Santiago et al., 2023; Chronicle of Higher Education, 2020). However, researchers did not investigate if similar findings occurred among university staff. In addition, studies on religiosity and mental health have primarily occurred outside the context of the pandemic (DeRossett et al., 2021; Jansen et al., 2010; Rahman et al., 2024). Therefore, a gap remains in understanding of the pandemic-related impact on university staff, and how religiosity was related to the coping of students, faculty, and staff (Meeks et al., 2021). This study examined the relationship between religiosity and several mental health outcomes (burnout, depression, and anxiety) among university students, staff, and faculty during the COVID-19 pandemic at a single, faith-based university. A better understanding of these relationships may help in the development of targeted interventions and support strategies to improve mental health and well-being within the university community.

The present study has three aims. First, to examine the prevalence of depression, anxiety and burnout among university faculty, staff, and students. Second, to explore the relationship between depression, anxiety and burnout with religiosity. Third, to explore predictors of depression and anxiety in three separate models of organized religious activity, private religious activity, and intrinsic religiosity among staff, faculty and students. Some of these results have been previously presented in a preliminary abstract (Yohannes & Williams, 2022).

## Methods

### Study Design

This study employed a cross-sectional anonymous online survey design at a single faith-based university in the USA. The University Communications Department distributed the online survey questionnaire to students, while the Institutional Research Office distributed the survey to faculty, and the Staff Council Office distributed the survey questionnaire to staff. Two reminders were sent out during the study period. As faculty members at this university, the investigators were uninvolved in questionnaire distribution to avoid bias and coercion in participation. Data were collected between November 2021 and April 2022, during the heart of the pandemic.

### Participants

The sample comprised a convenience sample of 125 participants (faculty, staff, and students). Eligible criteria: participants were aged > 18 years old and classified as an employee and enrolled as a student at the university. Persons who were under the age of 18 years old, non-university employees, or inactive students were exempt from the study. Individual-level attributes such as race/ethnicity, age, gender, and relationship status were assessed during the survey. Submission of survey responses would take under twenty minutes to complete.

### Ethical Considerations

The study was approved by the university research ethics committee (Ref: 21-234). This study was conducted in line with the Declaration of Helsinki. Completion of the survey was assumed to represent informed consent, ensuring voluntary participation and protecting participants’ rights and privacy.

### Outcome Measures

The survey consisted of three distinct measures, including the 5-item Duke University Religion Index (DUREL), the Hospital Anxiety and Depression Scale (HADS), and the Oldenburg Burnout Inventory (OLBI).

#### Duke University Religion Index (DUREL)

Self-reports of religiosity were measured using the Duke University Religion Index (DUREL) (Koeing et al., 1997). This five-item scale has three subscales: organizational religious activity (ORA), non-organizational religious activity (NORA), and intrinsic religiosity (IR). ORA is assessed as, “How often do you attend church or religious meetings? (scored 1= Never to 6= more than once a week). NORA is assessed as, “How often do you spend time with private religious activities such as prayer or bible reading?” (scored 1= rarely or never to 6= more than once a day). The three IR statements reflecting personal commitment and motivation are: “In my life I experience the presence of God”; “My religious beliefs are what really behind my whole approach to life”; and “I try hard to carry my religion over into all other dealings in life.” These statements are scored 1= definitely not true of me, to 5= definitely true of me. Aggregate scores for IR range from 3 to 15. High scores on ORA and NORA have been related to better health and positive health behaviors (Koenig et al., 1997). The DUREL was established as a valid and reliable instrument to assess religiosity, both as a single composite score and as three subscale scores (Koenig et al., 1997; Lace & Handal, 2018; Storch et al., 2004a, 2004b). Cronbach’s alpha coefficient for DUREL range from 0.78 to 0.91 (Koenig & Bussing, 2010).

#### Hospital Anxiety Depression Scale (HADS)

HADS consists of fourteen items with two subscales: HADS-anxiety (HADS-A) and HADS-depression (HADS-D). Each subscale is made up of seven items with a 4-point response format. Scores range from 0 to 21 for each subscale, with a cut-off score HADS-A ≥ 8 and HADS-D ≥ 8, which indicate clinically relevant anxiety and depressive symptoms that require medical intervention (Zigmond et al., 1983; Bjelland et al., 2002). Bjelland et al. (2002) systematic review concluded that the HADS is a valid tool in screening for anxiety and depression in patients with chronic diseases and community populations. The Cronbach alpha coefficients for HADS range from 0.78 to 0.93 for the anxiety subscale and from 0.82 to 0.90 on the depression subscale (Velligan et al., 2002).

#### Oldenburg Burnout Inventory Scale (OLBI)

The OLBI is a 16-item scale measuring burnout with two subscales: exhaustion defined as (physical, emotional, and cognitive fatigue) and disengagement characterized by negative attitudes toward work objects, work content or work in general (Halbesleben & Demerouti, 2005). Each subscale has eight questions and response options ranges across a 4-point Likert scale from “strongly disagree” to “strongly agree.” To calculate the total burnout score, instructions are to average all 16 items; for the subscales disengagement and exhaustion, they too are average across all 8 items per subscale. Cut-off scores indicating burnout severity are the following with the mean scores: Exhaustion scores > 2.25 indicates “high exhaustion,” Disengagement scores > 2.10 reveals “high disengagement,” and a total burnout score > 2.18 indicates “high overall burnout” (Demerouti & Bakker, 2008). Halbesleben and Demerouti (2005) established that the English translation of the OLBI is a reliable and valid tool for measuring burnout. The Cronbach alpha coefficients for OLBI range from 0.74 to 0.87 for the exhaustion subscale and from 0.70 to 0.83 on the disengagement subscale (Demerouti et al., 2003; Halbesleben & Demerouti, 2005).

### Data Analysis

Descriptive statistics summarize sociodemographic and outcome measures using frequencies, percentages, means, and standard deviations. The Chi-square test was performed for categorical variables. Comparison between group means was by analysis of variance. The relationship between DUREL (ORA, NORA, and IR scores) versus psychological measures, burnout variables, age, gender and marital status were assessed using the Pearson correlation analysis for continuous variables when normal distribution conditions were met and Spearman rho correlation analysis when normal distributions were not met. Performed were two univariate logistic regression analyses using the HAD-depression ≥ 8 versus < 8 and HAD-anxiety ≥ 8 versus < 8 as “dependent variables” with variables that are correlated with DUREL. Variables that showed statistically significant associations (*p* < 0.05) were entered into the three models (Model 1, ORA; Model 2, NORA and Model 3, IR) controlling confounding factors of age, gender, and educational status. ORA, NORA and IR were examined separately using logistic multiple regression analyses to avoid collinearity. Data were analyzed with the Statistical Package for the Social Sciences (SPSS, version 27.0™). Significance was set at *p* < 0.05.

## Results

A total of 125 respondents completed the survey: 17 faculty, 18 staff, and 90 students. The respondents’ mean age was 30 (SD = 12.3) years, with 69% female. Faculty were older, with mean age 51 (SD = 12.8) years, while staff were 39 (SD = 11.8) and students 25.0 (SD = 5.5) years [*F* = 90.38, *p* < 0.001]. Faculty had lower mean depression symptoms 3.82 (SD = 3.30), staff 6.78 (SD = 3.90), and students 6.50 (SD = 4.0) [*F* = 3.5, *p* = 0.03] and anxiety symptoms 5.47 (SD = 2.91) versus 8.94 (SD = 3.6) versus 9.81 (SD = 4.5), respectively [*F* = 7.42, *p* < 0.001]. There were no statistically significant differences between students and staff in anxiety and depressive symptoms. The sociodemographic characteristics of the participants are illustrated in Table 1.

**Table 1:** Sociodemographic characteristics and outcome measures (*n* = 125)

Variables	Mean	SD
Age in Years	30	12
HAD-depression	6	4.21
HAD-anxiety	9	4.36
	*Number*	%
*Participants*		
Faculty Staff Students	17 18 92	14.0 14.0 72.0
*Education level*		
Bachelor of Art/ Bachelor of Science Master’s Degree Doctoral Degree	59 13 53	47.0 10.0 43.0
*Gender*		
Male Female Prefer not to say	37 86 2	30.0 69.0 1.0
HADS-anxiety ≥ 8	78	62.0
HADS-depression ≥ 8	51	41.0
*Burnout severity using OLBI*		
Exhaustion > 2.10 Disengagement > 2.20 Total burnout > 2.18	40 91 66	32.0 73.0 53.0
*Marital status*		
Single Married Domestic partnership Divorced	77 45 3 1	62.0 35.0 2.0 1.0
*Race/Ethnicity*		
White Asian Americans Spanish Americans Others Black African Americans	64 26 16 12 6	51.0 21.0 13.0 10.0 5.0

HADS, Hospital anxiety depression scale

SD, Standard deviation

OLBI, Oldenberg Burnout Index

### Religious Attendance, Private Religious Activities, and Intrinsic Religiosity

Sixty-one participants (49.0%) attended a church service at least once or more per week, and 46 (37.0%) spent time in religious activities (e.g., reading the bible and prayer) each day, and 48 (38.0%) spending time in religious activities one or more times per week. Over 92.0% of the participants experienced the presence of the divine God in their lives, over 89.0% felt their religious beliefs underlie their whole approach to life, and over 84.0% carry their religion into all other dealings of life (Table 2).

**Table 2:** Religious activities, and attitudes (*n* = 125)

Religious activities	N	%
*Organizational religious activity (ORA) (e.g., public religious activities)*		
Never	17	13.0
Once a year or less	11	9.0
A few times a year	18	14.0
A few times a month	18	14.0
Once a week	35	28.0
More than once a week	26	21.0
*Non-organizational religious activity (NORA) (e.g., private prayer, bible study)*		
Rarely or never	32	26.0
Daily	37	30.0
More than once a day	9	7.0
Once a week	14	11.0
Two or more times a week	22	18.0
More than once a week	26	21.0
A few times a month	11	9.0
*Intrinsic religiosity (IR) (e.g., personal religious commitment or motivation* [true or tends to be true]		
In my life, I experience the presence of the divine God Mean (SD)	92 3.96	74.0 1.11
My religious beliefs are what really lie behind my whole approach to life Mean (SD)	89 3.94	71.0 1.27
I try hard to carry my religion over into all other dealings of life Mean (SD)	84 3.79	67.0 1.35

Table 3 shows ORA was negatively associated with depression (*r* = − 0.23, *p* < 0.009), anxiety (*r* = − 0.28, *p* < 0.002). Private religious activity (bible reading and prayer) correlated positively with older age (*r* = 0.22, *p* = 0.01), being married (*r*_s_ = − 0.24, *p* < 0.006) and negatively related with depression (*r*_s_ = − 0.22, *p* = 0.01) and anxiety (*r*_s_ = − 0.36, *p* < 0.001). IR was correlated positively with older age (*r* = 0.29, *p* = 0.001), being married (*r*_s_ = − 0.26, *p* < 0.003) and higher level of education (*r*_s_ = 0.20, *p* = 0.02); and negatively correlated with depression (*r* = − 0.38, *p* < 0.001) and anxiety (*r* = − 0.37, *p* < 0.001).

**Table 3:** Relationship of Duke University Religious Index (DUREL) with sociodemographic and clinical variables

	Age	Marital status	Depression	Anxiety	Total burnout	Gender	Education level
ORA	*r* = 0.22, *p* = 0.01	*r*_s_ = − 0.17, *p* = 0.06	*r* = − 0.23, *p* < 0.009	*r* = − 0.28, *p* < 0.002	*r* = − 09, *p* = 0.32	*r*_s_ = 0.06, *p* = 0.46	*r*_s_ = 0.05 *p* = 0.56
NORA	*r* = 0.32, *p* < 0.001	*r*_s_ = − 0.24, *p* < 0.006	*r* = − 0.23, *p* = 0.01	*r* = − 0.37, *p* < 0.001	*r* = − 15, *p* = 0.09	*r*_s_ = 0.10, *p* = 0.25	*r*_s_ = 0.19 *p* = 0.03
IR	*r* = 0.29, p < 0.001	*r*_s_ = − 0.28, *p* < 0.002	*r* = − 0.38, *p* < 0.001	*r* = − 0.37, *p* < 0.001	*r* = − 14, *p* = 0.13	*r*_s_ = 0.07, *p* = 0.42	*r*_s_ = 0.20 *p* = 0.02

Marital status (1 = married, 2 = single)

ORA, organizational religious activity

NORA, non-organizational religious activity

IR, intrinsic religiosity (sum of the three intrinsic religiosity measures)

Total burnout (sum of disengagement plus exhaustion)

Table 4 shows the univariate predictors of depression determined by HADS-depression ≥ 8. Unmarried participants were twice as likely to exhibit depression (*p* < 0.009) as married individuals. Regular attendees of organized religious activities were 34% less likely to exhibit depression (*p* < 0.001). Participants engaging in private religious activities were 27% less likely to exhibit depression (*p* < 0.006). Those experiencing IR were 23% less likely to exhibit depression (*p* < 0.001). Participants experiencing burnout were 10 times more likely to exhibit depression (*p* < 0.003).

**Table 4:** Univariate analysis of elevated depression associated with religiosity and total burnout

Variable	Univariate analysis HAD-depression < 8 versus HAD-depression ≥ 8	
	Odds ratio 95% confidence interval	*p*-value
Marital status	2.63 (1.27–5.44)	< 0.009
ORA	0.66 (0.52–0.83)	< 0.001
NORA	0.73 (0.59–0.92)	< 0.006
IR	0.77 (0.69–0.87)	< 0.001
Educational status	1.05 (0.62–1.81)	= 0.83
Age	0.97 (0.94–1.01)	= 0.10
Gender	1.53 (0.75–3.10)	= 0.24
Total Burnout	10.08 (2.23–45.70)	< 0.003

Marital status (1 = married, 2 = single)

ORA, organizational religious activity

NORA, non-organizational religious activity

IR, intrinsic religiosity

Table 5 shows the univariate predictors of anxiety determined by HADS-anxiety ≥ 8. Unmarried participants were twice as likely to exhibit anxiety (*p* = 0.01) compared to married individuals. Regular attendees of organized religious activities were 24% less likely to exhibit anxiety (*p* = 0.01), those engaged in private religious activities were 27% less likely to exhibit anxiety (*p* < 0.007), and participants experiencing IR were 20% less likely to exhibit anxiety (*p* < 0.001). Older participants were 4% less likely to exhibit anxiety symptoms compared to younger people (*p* < 0.008). Lower educational status correlated with being 48% more likely to exhibit anxiety (*p* = 0.02). Participants experiencing burnout are eleven times more likely to exhibit anxiety (*p* < 0.003).

**Table 5:** Univariate analysis of elevated anxiety associated with religiosity, younger age and total burnout

Variable	Univariate analysis HAD-anxiety < 8 versus HAD-anxiety ≥ 8	
	Odds ratio 95% confidence interval	*p*-value
Marital status	2.26 (1.18–4.35)	= 0.01
ORA	0.76 (0.60–0.95)	= 0.02
NORA	0.73 (0.58–0.91)	< 0.007
IR	0.80 (0.70–0.92)	< 0.001
Educational status	0.52 (0.30–0.92)	= 0.02
Age	0.96 (0.934–0.99)	< 0.008
Gender	1.53 (0.31–1.27)	= 0.19
Burnout	11.53 (2.31–57.47)	< 0.003

Marital status (1 = married, 2 = single)

ORA, organizational religious activity

NORA, non-organizational religious activity

IR, intrinsic religiosity

The models were rerun using multiple logistic regression to assess the effects of controlling age, gender and educational status on anxiety as shown in Table 6. Model 1 for ORA excludes NORA and IR to avoid collinearity. Participants attending church services regularly were 22% less likely to exhibit anxiety (*p* = 0.04) and those experiencing burnout were nine times more likely to exhibit anxiety (*p* < 0.007). The trend for unmarried participants to exhibit anxiety did not reach statistical significance (*p* = 0.06). In Model 2 for NORA, regular engagement in private religious activities reduced the likelihood of exhibiting anxiety by 23% (*p* = 0.03), while participants experiencing burnout were eight times more likely to exhibit anxiety (*p* = 0.01). Like Model 1 for ORA, there was a trend for unmarried participants to exhibit anxiety (*p* = 0.07). In Model 3 for IR, participants experiencing the presence of divine God were 17% less likely to exhibit anxiety (*p* < 0.009). Participants experiencing burnout were eight times more likely to exhibit anxiety (*p* = 0.01).

**Table 6:** Multivariate analysis of elevated anxiety was associated with religiosity and total burnout

Variables	Multivariate analysis HAD-anxiety < 8 versus HAD-anxiety ≥ 8					
	Model 1—ORA*		Model 2—NORA*		Model 3—IR*	
	Odds ratio 95% confidence interval	*p*-value	Odds ratio 95% confidence interval	*p*-value	Odds ratio 95% confidence interval	*p*-value
ORA	0.78 (0.61–0.99)	= 0.04	-	_	_	_
NORA	–	-	0.77 (0.60–0.98)	= 0.03	_	_
IR	–	-	–	–	0.83 (0.73–0.96)	< 0.009
Marital status	1.92 (0.97–3.79)	= 0.06	1.86 (0.95–3.65)	= 0.07	1.67 (0.82–3.42)	= 0.15
Burnout	9.96 (1.85–53.71)	< 0.007	8.44 (1.58–45.01)	= 0.01	8.64 (1.61–46.28	= 0.01

ORA, organizational religious activity

NORA, non-organized religious activity

IR, intrinsic religiosity

(-) Signify not included in the model

Marital status = Married = 1, Single = 2

^*^Controlled for age, gender, educational status

Similarly, Table 7 shows the results of multiple logistic regression analysis controlling for age, gender and educational status on depression. In Model 1 for ORA, participants attending church services regularly were 33% less likely to exhibit depressive symptoms (*p* < 0.005). Those experiencing burnout were ten times more likely to exhibit depression (*p* < 0.006). Once again, there was only a trend for unmarried participants to exhibit higher levels of depression (*p* = 0.06). In Model 2 for NORA, participants regularly engaging in private religious activities were 22% less likely to exhibit depressive symptoms (*p* = 0.04) and participants experiencing burnout were seven times more likely to exhibit depression (*p* = 0.01). Unmarried participants were twice as likely to exhibit depression (*p* = 0.05). In Model 3 for IR, participants experiencing intrinsic religiosity were 21% less likely to exhibit depression (*p* < 0.001) and participants experiencing burnout were eight times more likely to exhibit depression (*p* = 0.01). There was no statistically significant relationship between marital status and depression.

**Table 7:** Multivariate analysis of elevated depression was associated with religiosity and total burnout

Variables	Multivariate analysis HAD -depression < 8 versus HAD-depression ≥ 8					
	Model 1—ORA*		Model 2—NORA*		Model 3—IR*	
	Odds ratio 95% confidence interval	*p*-value	Odds ratio 95% confidence interval	*p*-value	Odds ratio 95% confidence interval	*p*-value
ORA	0.67 (0.53–0.86)	< 0.001	_	_	_	_
NORA	_	_	0.78 (0.62–0.99)	= 0.04	_	_
IR	_	_	_	_	0.79 (0.70–0.91)	< 0.001
Marital status	2.02 (0.96–4.26)	= 0.06	2.09 (1.00–4.38)	= 0.05	1.76 (0.81 – 3.84)	= 0.15
Burnout	10.08 (1.92–52.83)	< 0.006	7.59 (1.61–35.73)	= 0.01	8.37 (1.68–41.78)	= 0.01

ORA, organizational religious activity

NORA, non-organized religious activity

IR, intrinsic religiosity

(-) Signify not included in the model

Marital status = Married = 1, Single = 2

^*^Controlled for age, gender, educational status

### Burnout, Depression, Anxiety, and Religiosity

Burnout has two subsections (disengagement and exhaustion) which were scored separately and summed up to obtain a total burnout score. Among our participants, high disengagement was associated with depression (*r* = 0.36, *p* < 0.001), anxiety (*r* = 0.38, *p* < 0.001), and with singleness (unmarried status) (*r*_s_ = 0.22, *p* = 0.01). The total burnout scores also correlated with depression (*r* = 0.31, *p* < 0.001), and anxiety *r* = 0.33, *p* < 0.001. Exhaustion was not associated with depression or anxiety symptoms (all *p* > 0.05). Younger age correlated with disengagement (*r* = 0.21, *p* = 01), total burnout (*r* = 0.19, *p* = 0.03), and anxiety (*r* = 0.23, *p* = 0.01). Exhaustion was not associated with ORA, NORA and total IR (all *p* > 0.05). My religious beliefs are what really lie behind my whole approach to life was negatively associated with disengagement (*r* = − 0.18, *p* = 0.04). and a trend to total burnout (*r* = − 0.15, *p* = 0.09). Disengagement was not associated with NORA or ORA or total IR (all *p* > 0.05).

## Discussion

The findings of this study highlight several unique points. First, frequent church attendance (organized religious activity, ORA), spending more time in private religious activities (NORA), and experiencing (intrinsic religiosity, IR) were all associated with less depression and anxiety. Second, being married was associated with frequent church attendance and engaging in both private and intrinsic religious activities. Finally, total burnout was a powerful indicator in multivariate regression analyses of interactions between public and private religiosity, and IR. Total burnout scores significantly increased with higher risk (odds) of experiencing anxiety and depression.

Over two-thirds of individuals exhibited symptoms of depression and three fifths experienced anxiety during the pandemic. These prevalences are significantly higher than previously reported in higher educational, non-pandemic multi-religious settings such as the 5% and 17% frequency of depression and anxiety, respectively, reported by Francis et al. (2019). The unprecedented impact of the pandemic gripped the survey participants in fear and social isolation; and the risk of contracting COVID-19 in turn exacerbated stress, fatigue and mental health disorders (Yohannes, 2020). Notably, the findings highlight the need to screen for depression and anxiety, especially in university students and staff and devise appropriate treatment interventions.

A wealth of evidence in both cross-sectional and longitudinal studies suggests that weekly religious attendance promotes better mental well-being, reducing both anxiety and depression (Kidwai et al., 2014; Koenig et al., 2020). In accord, with those findings, our study adds to that evidence base by demonstrating the protective effect of religiosity on mental well-being. During the unprecedented pandemic, regular weekly church attendance was associated with reduced depression and anxiety symptoms. These findings highlight the value and benefits of regular, public religious gatherings. In church, individuals might derive significant benefits from encouragement, camaraderie of friendship and emotional support from church members and pastors that may promote better coping mechanisms and improve quality of life (Burges et al., 2021). In addition, the church served as a sanctuary during the pandemic, amid unparalleled fear of contracting COVID-19. Church attendance fostered community interactions, providing social support with like-minded people and positive coping mechanisms through shared communal prayers, beliefs and faith in the higher power of deity.

Regularly engaging in private religious activities (e.g., daily bible reading and prayer) was associated with fewer anxiety and depression symptoms compared to those with few or no spiritual activities, as shown in Tables 6, 7. Furthermore, those engaged with a high level of spirituality tend to attend religious services more frequently than those with lower levels of spirituality. Regular spiritual activity and spending time in regular worship and devotion has potential benefits to install peace, comfort and increased faith in deity, which in turn may produce better coping mechanisms and engender fewer anxiety and depression symptoms.

An interesting finding in our study was the association of IR with fewer symptoms of depression and anxiety. These findings are unique compared to others that religiosity, with prayer and reading scriptures, in times of pandemic and other negative life events provide peace and comfort. Certainly, actively seeking comfort and blessings from God has correlated with better psychological well-being and coping, while not relying on God related to poor psychological adjustment and coping mechanisms (Ano et al. 2005). However, caution is required in interpreting our survey findings as we did not examine individuals’ coping mechanisms.

Being married and older were associated with lower odds of exhibiting depression and anxiety symptoms. Older participants were more likely to engage in private reading the bible and prayers and intrinsic religious activities compared to single individuals. A recent systematic review and meta-analysis showed that older people engaged in religious and spiritual practices had greater life satisfaction, better psychological well-being and social relations, and found more meaning in life (Coelho-Junior et al., 2022).These findings are also previously reported in older adults with chronic diseases and mental health disorders (Koenig et al., 2020). It is possible that NORA and IR may provide a favorable environment for spouses to engage together in reading the bible and praying, which in turn promotes intimate relationships. Our data highlight that approaching life according to belief in the presence of the divine was associated with fewer anxiety and depressive symptoms (Tables 6, 7).

Participants with high total burnout scores were ten times more likely to exhibit depression and anxiety compared to those without burnout in both models of ORA, and eight times more likely for both NORA and IR (Tables 6, 7). Burnout scores were also a consistent predictor of anxiety and depression among our participants. This is unsurprising given the significant positive correlation between both depression and anxiety with burnout in our univariate correlations (Table 4). However, caution is necessary as the confidence intervals for these observations are very wide, and replication in a larger prospective study is required.

In our study, staff exhibited higher levels of anxiety and depression comparable to students, but less than faculty. In contrast, a previous study found similar levels of anxiety and depression in staff, faculty and student populations (Meeks et al., 2021). Thus, there is a need for further investigation of university staff and students to identify supportive resources and foster effective coping mechanisms to enhance a healthier work and studying environment. A recent systematic review and expert consensus report highlighted the importance of addressing spirituality in serious illness and health outcomes as part of person-centered and value sensitive care (Balboni et al., 2022).

### Implications for Higher Institution Administrators and Religious Leaders

The impact of pandemic was pervasive in higher institutions with unprecedented stress with exceedingly high mental health issues that affected faculty, staff and students. Thus, if resources are available, university administrators, in collaboration with counselors and chaplains, might consider addressing mental health issues now or in a future pandemic:

- Develop strategies that foster a collaborative approach with university counselors and chaplains that connect individuals to culturally competent care during high stress periods.
- Create safe spaces and environments for individuals with elevated levels of stress, depression and anxiety through campus ministries to foster belonging through informal gatherings, shared meals, and social activities to reduce social isolation.
- Have university administrators provide dedicated, accessible, and quiet spaces for silence, prayer, and meditation for all religions, along with exploring how to encourage peer-to-peer support groups.
- Train chaplains and religious leaders to recognize mental health issues and warning signs that might prompt referral to professional counseling services when appropriate.
- Offer religious services in university campuses both in-person and live streamed. Such hybrid access allows students, staff and faculty to connect with their faith community (e.g., weekly or mid-week), regardless of geographic location. Participation in religious activities will foster holistic mental well-being and promote resilience and active engagement in a faith community.

## Limitations

There are several limitations of the study. First, the sample size is relatively small and is a sample of convenience. The low response rate raises concerns that the respondents did not adequately represent the demographic composition of either the staff or the faculty. Differences in response rates between faculty, staff, and students might have introduced unwanted biases among the responders. Furthermore, the small numbers of faculty and staff responders relative to number of students who responded precludes a direct comparison between the three groups. Second, we did not collect data on religious affiliation, psychosocial factors such as financial stressors, comorbidities, or adverse chronic diseases which might affect individual well-being. Third, causation cannot be assumed from this cross-sectional study. Further work is required in a large sample size to examine the benefits of religiosity in a prospective longitudinal study on mental health outcomes. In addition, future studies should explore the mechanisms and pathways by which religious activities might influence mental health. Fourth, we employed self-reported validated depression and anxiety scale. Ideally, it would have been useful to conduct clinical interviews using a diagnostic tool for major depression and anxiety consistent with the Diagnostic and Statistical Manual of Mental Disorders (5^th^ edition) criteria. Fifth, we did not collect data on whether participants were on anti-depressants and/or anxiolytics symptoms. Finally, the study was conducted at a single Christian university in the USA. Thus, it requires replication for generalizability in non-Christian-based public and private higher education institutions.

## Conclusions

A higher frequency of weekly religious attendance, engaging in meaningful spiritual activities in daily basis and religious beliefs on deity were associated with less anxiety and depression in adults during the COVID-19 pandemic. These findings remained constant both in univariate and multivariate analyses when demographic factors (age, gender and race) were held constant. Burnout was associated with elevated symptoms of depression and anxiety symptoms in university students, staff and faculty. Further work to examine the beneficial effects of religion and spiritual intervention with and without lifestyle interventions (e.g., structured exercise program) in prospective, controlled trials to ameliorate anxiety and depression would be worthy endeavors.
